# How Patient-Generated Data Enhance Patient-Provider Communication in Chronic Care: Field Study in Design Science Research

**DOI:** 10.2196/57406

**Published:** 2024-09-10

**Authors:** Dario Staehelin, Mateusz Dolata, Livia Stöckli, Gerhard Schwabe

**Affiliations:** 1 Department of Informatics University of Zurich Zurich Switzerland; 2 Department for Information and Process Management Eastern Switzerland University of Applied Sciences St Gallen Switzerland

**Keywords:** patient-provider communication, patient-generated data, field study, chronic care, design science research, patient-centered care, integrated care, patient-provider collaboration, mobile phone

## Abstract

**Background:**

Modern approaches such as patient-centered care ask health care providers (eg, nurses, physicians, and dietitians) to activate and include patients to participate in their health care. Mobile health (mHealth) is integral in this endeavor to be more patient centric. However, structural and regulatory barriers have hindered its adoption. Existing mHealth apps often fail to activate and engage patients sufficiently. Moreover, such systems seldom integrate well with health care providers’ workflow.

**Objective:**

This study investigated how patient-provider communication behaviors change when introducing patient-generated data into patient-provider communication.

**Methods:**

We adopted the design science approach to design PatientHub, an integrated digital health system that engages patients and providers in patient-centered care for weight management. PatientHub was developed in 4 iterations and was evaluated in a 3-week field study with 27 patients and 6 physicians. We analyzed 54 video recordings of PatientHub-supported consultations and interviews with patients and physicians.

**Results:**

PatientHub introduces patient-generated data into patient-provider communication. We observed 3 emerging behaviors when introducing patient-generated data into consultations. We named these behaviors *emotion labeling*, *expectation decelerating*, and *decision ping-pong*. Our findings show how these behaviors enhance patient-provider communication and facilitate patient-centered care. Introducing patient-generated data leads to behaviors that make consultations more personal, actionable, trustworthy, and equal.

**Conclusions:**

The results of this study indicate that patient-generated data facilitate patient-centered care by activating and engaging patients and providers. We propose 3 design principles for patient-centered communication. Patient-centered communication informs the design of future mHealth systems and offers insights into the inner workings of mHealth-supported patient-provider communication in chronic care.

## Introduction

### Background

The quality of the patient-provider relationship is strongly linked to patients’ increased adherence and better health outcomes [1-3]. Patient-provider communication requires exchanging accurate and relevant information to better understand patients and their preferences and context [4,5]. However, provider instructions are complex and not communicated adequately to patients [3,6] as health care providers often lack the time or communication training [7]. In turn, patients have difficulties recalling crucial information (eg, their adherence to taking medication regularly), impeding providers’ ability to quickly assess their medical condition and derive actions [8,9]. Therefore, adherence and health outcomes are often subpar—especially in people with chronic conditions [6,10,11]. Due to its centrality, improving patient-provider communication is a topic of continued interest in medical research.

### Patient-Centered Care

Over the last decades, the understanding of good patient-provider communication has evolved. Historically, providers have possessed most of the power in patient-provider communication [12]. These power dynamics mainly occurred due to the significant knowledge difference between providers and patients [13]. The resulting paternalistic model, in which the providers made all the decisions, led to poor adherence and increased health care costs [14,15]. Newer approaches such as shared decision-making and patient-centered care ask providers to adopt more inclusive methods focusing on the collaborative nature of patient-provider communication [4,5,16-18].

**Figure 1:**
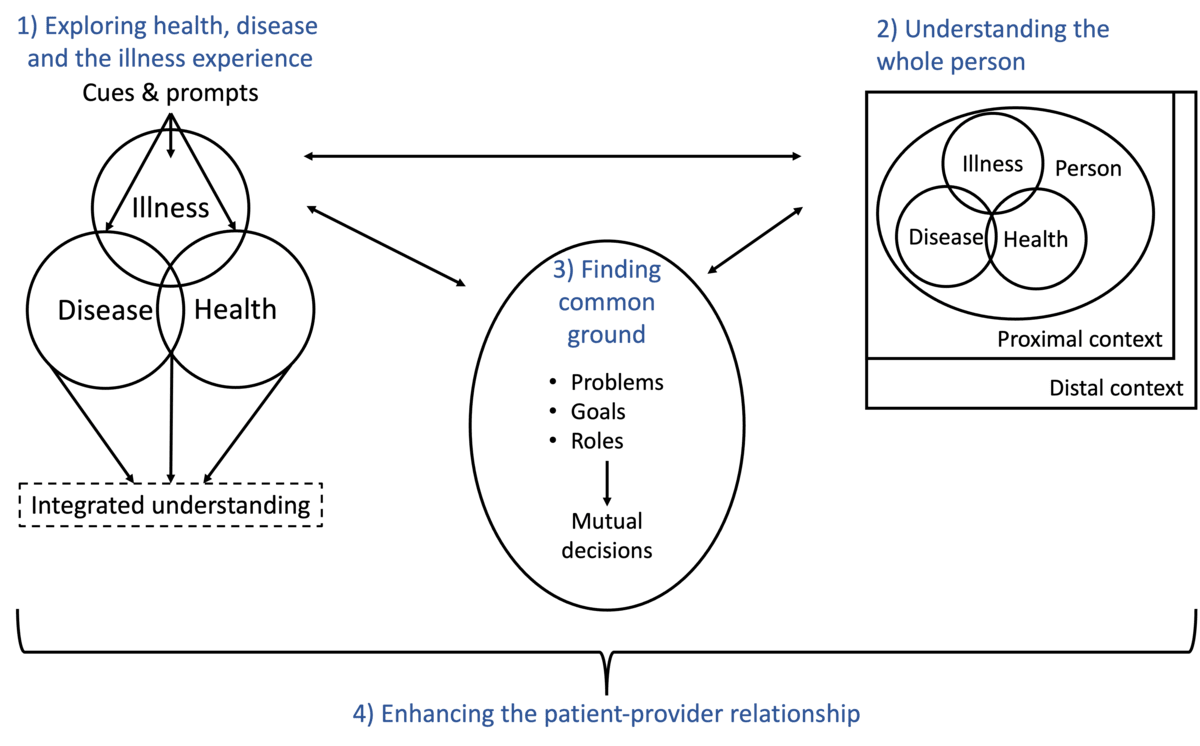
Patient-centered care framework by Stewart et al [4].

Patient-centered care proposes a holistic clinical method that centers on the patients and their preferences and contexts [4,5,19]. It is defined as “respectful of and responsive to individual patient preferences, needs, and values and ensuring that patient values guide all clinical decisions” and is 1 of 6 key elements of high-quality care [16]. The clinical method of patient-centered medicine by Stewart et al [4] is among the most frequently used frameworks. It proposes a communication approach that suggests looking beyond a patient’s acute problem and into their history and context (Figure 1). The framework suggests (1) *exploring health, disease, and the illness experience*; (2) *understanding the whole person*; and (3) *finding common ground* to (4) *enhance the patient-clinician relationship*. In the following paragraphs, we describe each of those dimensions.

Exploring health, disease, and the illness experience highlights the importance of understanding the patient’s experience. People have different interpretations and unique experiences of health and illness. Someone with an asymptomatic disease may perceive themselves as healthy, whereas others feel ill without having a disease. Providers should seek to understand and support the patient’s view of their situation and their experience of health and illness by listening to their concerns and feelings [4,20]

Understanding the whole person focuses on understanding the patient’s proximal and distal context [4]. It enables meaningful conversations about the illness and treatment options as providers know their patients as people (eg, what is currently important to them) [20]. Younger patients might struggle more with a diagnosis than older people. A patient’s relationships, work, education, lifestyle, and culture play a significant role in their treatment.

Patients and providers work together to *find common ground* regarding the problems and priorities, the goals of the therapy, and the roles of the patient and the provider [4]. It stresses the emotional engagement with the patient and the genuinely collaborative aspect of finding common ground to arrive at mutual decisions [4].

Enhancing the patient-clinician relationship is the goal of every encounter in patient-centered medicine [4]. Health care becomes genuinely patient centered through an integrated understanding of the patient’s experience, understanding them as a whole person, and mutual decisions [20]. The framework by Stewart et al [4] conceptualizes patient-provider communication to achieve patient-centered care. It proposes a mindset that places patients at the center of clinical practice.

### Patient-Generated Data in Patient-Provider Communication

This shift in the mindset has attracted increasing interest in health informatics research that studies the effect of technology on patient-provider communication [21-27]. In face-to-face consultations, patient data generated on mobile health (mHealth) apps become increasingly important as they allow patients and providers to gain deeper insights into patients’ routines and adherence to therapy plans. Patient-generated data are health-related data gathered or created by patients, usually through wearables and mHealth [28]. Studies in health informatics and related fields have demonstrated the potential of patient-generated data to increase patient-provider communication [22,29,30]. mHealth allows patients to generate abundant health information, such as dietary patterns, emotional conditions, or objective measures such as blood pressure [22,23,31]. These data allow for insights into the patient’s health experience and journey unlike ever before [32,33].

mHealth-supported approaches have significantly improved patient-provider communication, adherence, and health outcomes in chronic care [34-36]. Studies have shown how these patient-generated data allow patients and providers to engage in collaborative sensemaking that improves decision-making [23,30,31,37]. It allows for deeper discussions about personal values [14,16] and improves patients’ understanding of their condition and treatment [38]. Furthermore, introducing patient-generated data into consultations affects the role dynamics of therapeutic sessions [34]. For example, sharing clinical notes shifts power in the patient-provider relationship [36]. Other studies report how sharing patient-generated data through mHealth leads to greater disclosure and better communication in consultations, resulting in better health outcomes [30,31].

These insights have been validated for different age groups [30,39,40] and chronic conditions (eg, chronic kidney disease) [21,25,35,41]. This previous research shows the positive impact of introducing patient-generated data into consultations on adherence and health outcomes [35,36]. For example, Vitger et al [35] describe the positive impact of generating data on a smartphone app on patient activation, communication confidence, and preparedness for decision-making in patients with schizophrenia.

**Figure 2:**
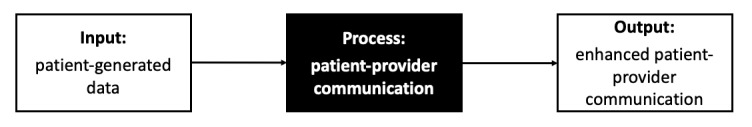
The process of patient-provider communication as a black box.

While existing research agrees on the vital role of patient-generated data in patient-provider communication [19,21,23], significant obstacles remain to leverage their potential. So far, structural and regulatory barriers have slowed advances [42,43]. mHealth apps seldom integrate with the provider’s workflow, leading to a fragmentation of health data [44,45]. More importantly, Cozad et al [46] found that only a few mHealth apps engage and activate patients to participate in patient-centered care. Finally, most studies report on the positive effects of patient-generated data on patient-provider communication, but they often fail to investigate the communication behaviors that use patient-generated data. Accordingly, patient-provider communication remains a black box that receives patient-generated data as input and creates better communication as output (Figure 2). Little to no research focuses on the design of systems that (1) integrate patient-generated data into the provider’s workflow and (2) use these data in consultations to enhance patient-provider communication. This study aimed to address this research gap by designing and evaluating an integrated digital health system that enhances the patient-provider communication.

## Methods

### Overview

This study addressed the research gap described previously by developing PatientHub in a design science research (DSR) approach to enhance the patient-clinician relationship [47,48]. DSR is a suitable approach as it systematically solves important general problems and generates new knowledge in the form of design principles, theoretical models, approaches, and impacts of technology use [49]. DSR proposes to ground a solution’s design in existing knowledge and theories, so-called kernel theories, to justify design decisions [50]. Due to these properties, design science is increasingly applied in medical informatics to study emerging technologies [48,51,52].

**Figure 3:**
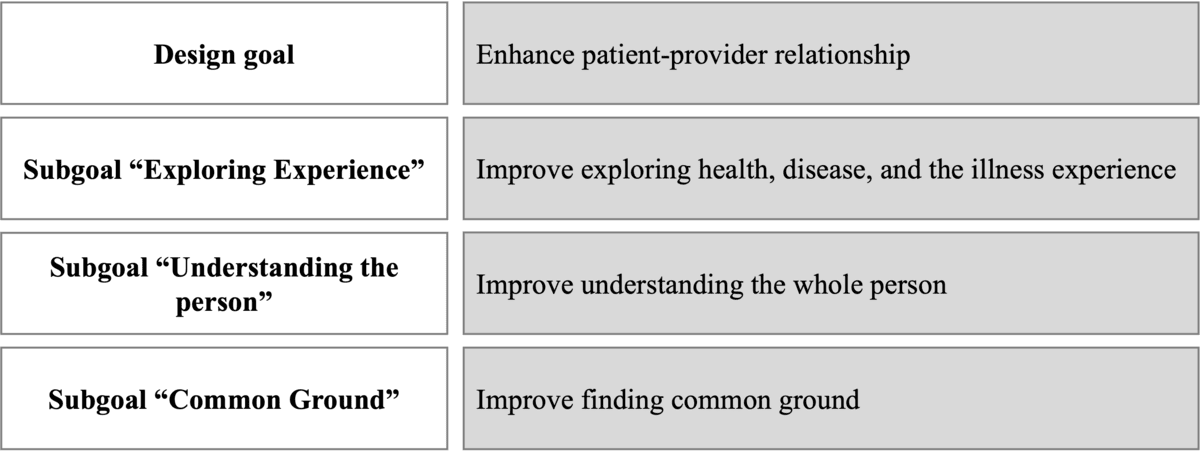
Design goal and subgoals based on the patient-centered care framework [4].

To address the research gap, we (1) designed PatientHub and (2) studied its impact on patient-provider communication by adopting the patient-centered care framework proposed by Stewart et al [4] as our kernel theory. PatientHub is an integrated digital health tool that introduces patient-generated data into consultations. We build on the strong correlation between patient-generated data and improved patient-provider communication established in recent work [34-36]. While the patient-centered care framework offers a holistic foundation for improving patient-provider communication, it lacks a clear operationalization of the 3 dimensions that offer mHealth designers and health care providers guidance on implementing patient-centered care. Specifically, it is unclear how to integrate patient-generated data into the consultation process and how patients and providers use them for patient-centered care. Accordingly, we formulated a design goal and several subgoals in line with our kernel theory (Figure 3 [4]).

The following sections present the PatientHub design, our field study approach, and the data analysis method.

### PatientHub Design

The project team designed PatientHub in 4 iterations. Our project team included a medical informatics company, a health institute specializing in chronic care, and 2 research institutions.

PatientHub’s design is grounded in the patient-centered care framework [4] and leverages existing design knowledge [21-23,34,39,53]. Over the 3 preliminary iterations, we continuously evaluated and improved the design. We tested the first design iteration by applying the think-aloud method with 7 participants acting as patients [54]. After refining the design in the second iteration, a focus group of 5 domain experts from software development, medicine, and research evaluated the revised PatientHub. In the third iteration, 3 health care providers and 5 patients evaluated the design in role-plays of consultations. This paper reports on the field study evaluating the prototype with actual patients and physicians.

PatientHub aims to enhance patient-provider communication by integrating patient-generated data into the consultation. It consists of a patient app, where patients generate data, and a consultation app, where patient-generated data provide a foundation for discussion in the consultation. In the following section, we describe the design implementation using a scenario and screenshots of PatientHub. It represents a potential user story of PatientHub during the field study.

### PatientHub Scenario

John is a patient at Laura’s clinic struggling with obesity. Last week, Laura proposed that John try PatientHub to help them advance John’s journey to better health. In the patient app on his smartphone, John tracked his dietary and activity habits in daily notes in a digital journal, filled out a general health questionnaire, and selected favorites for behavior change interventions as part of a 1-week preparation phase.

**Figure 4:**
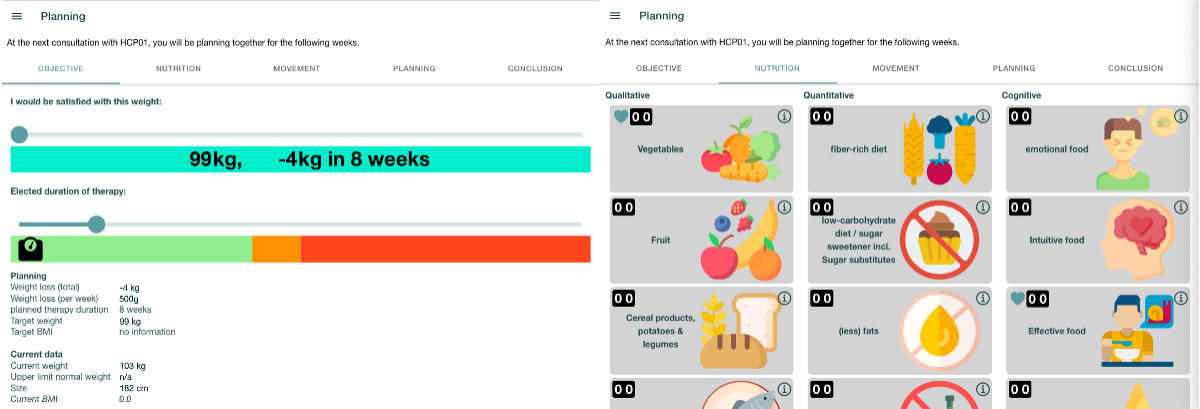
Goal setting (left) and dietary intervention (right) app screens.

In the initial consultation, Laura and John review the journal entries, questionnaire answers, and intervention favorites in the consultation app on a tablet. The consultation app consists of 4 screens: goal setting, defining dietary and activity interventions, planning, and closing. For goal setting, John and Laura discuss target weight and therapy duration with a tablet-based visualization using sliders (Figure 4, left). As the visualization relates weight loss and duration to each other, they can discuss healthy weight loss and set realistic goals. To define dietary and activity interventions, John liked 3 interventions he would like to explore. John and Laura can discuss additional interventions from a list of obesity-friendly interventions (Figure 4, right; selected by medical professionals).

**Figure 5:**
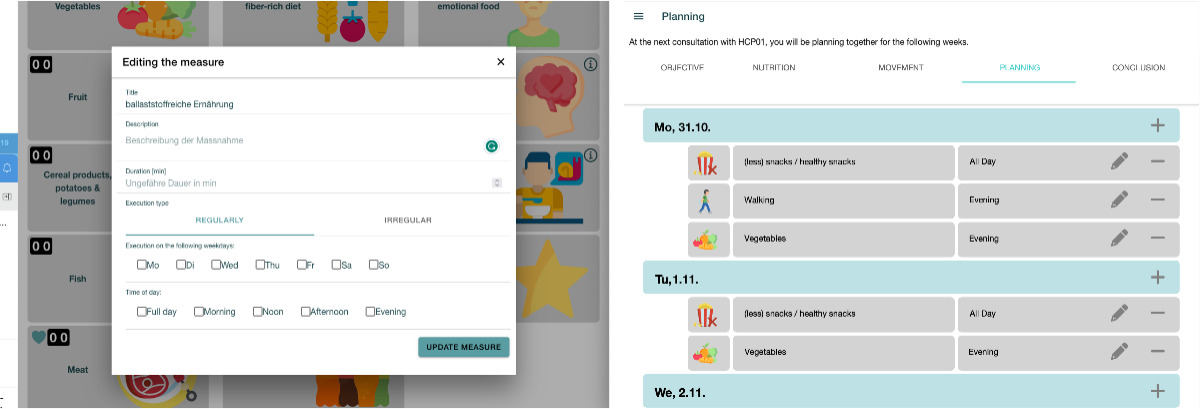
Measure specification (left) and planning (right) app screens.

To create an intervention, they specify a name, a description (eg, 8000 steps per day), a duration (if applicable), recurrence (ie, regular or irregular), and preferred days and times (Figure 5, left). The consultation app allows them to discuss the interventions to arrive at a patient-centered therapy plan considering John’s specific context. In planning, John and Laura see an overview of the interventions in calendar form (Figure 5, right). They can adjust the therapy plan if necessary (eg, move an intervention from Monday to Tuesday). Once the therapy plan is finalized, all information is automatically shared with John on the patient app.

**Figure 6:**
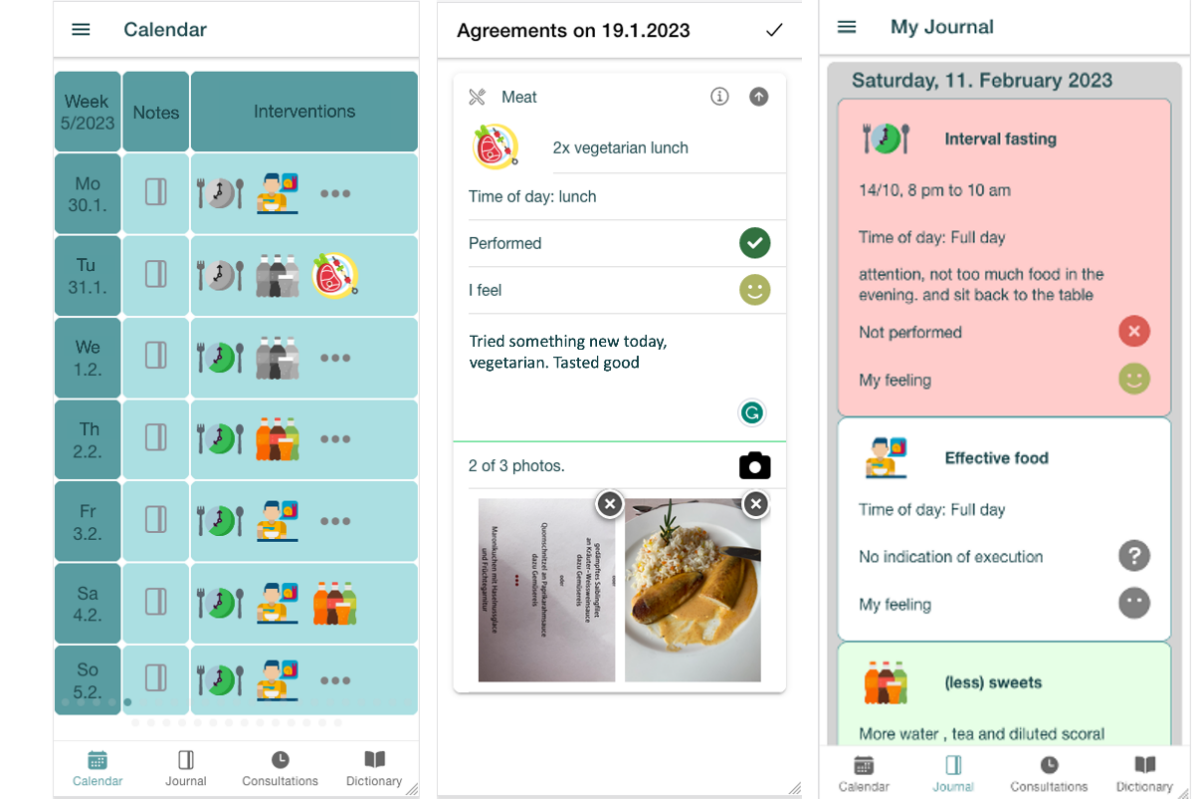
Calendar (left), journal entry (middle), and journal overview (right) app screens.

John tracks his health journey in PatientHub’s journal for 2 weeks. He tracks his adherence to the therapy plan and his experience while executing it. John sees each intervention in the calendar, where open interventions are grayed out and become colored once completed (Figure 6, left). For example, John had to limit carbohydrate-dense foods today. He clicks on the gray task icon to create a task-specific entry. He marks the task as completed and sets his emotional state to medium as he missed out on dessert today. John then uploads a picture of his lunch and writes a note (Figure 6, middle). He can now review his entries in his journal (Figure 6, right). John carried out the therapy plan and kept his digital journal during the 2-week implementation phase leading up to the follow-up consultation with Laura.

**Figure 7:**
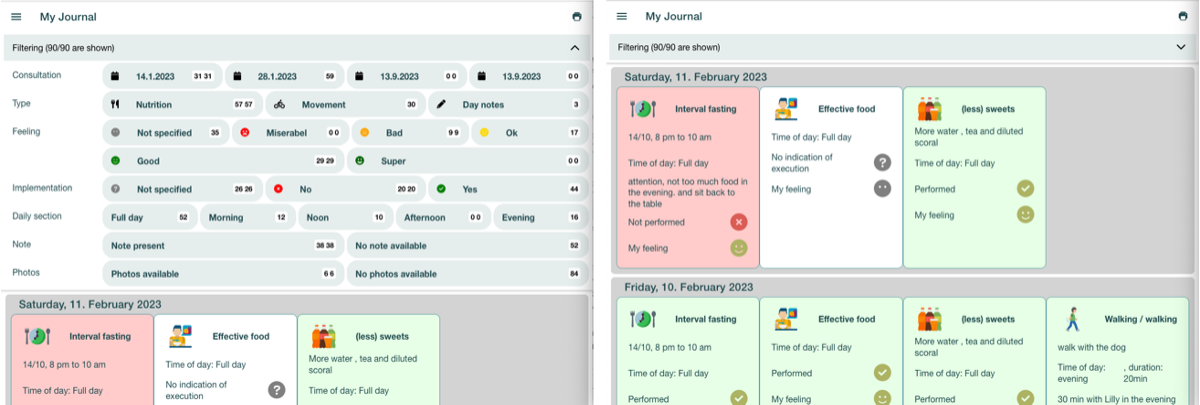
Filter options (left) and journal overview (right) app screens.

The follow-up consultation focuses on reviewing John’s journal. Laura and John apply different filters to the journal entries, such as task type (ie, diet, activity, and daily note), emotions, execution, and media type (Figure 7, left). This way, they can review interventions that were not completed or completed but not enjoyed by John (Figure 7, right). Through this discussion, they identify opportunities to improve the therapy plan and adherence. They adjust interventions as in the initial consultation by going through diet, activity, and planning before closing the consultation. Again, all data are shared across the PatientHub apps and the loop between consultations is closed. John enters a new implementation phase where he records his progress, which he will review again with Laura.

### Data Collection

#### Overview

In line with the “human risk and effectiveness” strategy [55], we evaluated PatientHub in a naturalistic setting to assess its effects on patient-provider communication. For this purpose, we collected data in a 3-week field study. The data set contained video recordings of each consultation and interviews with each participant after both consultations. We analyzed 27 initial and follow-up consultations (54 recordings) to study emerging behaviors when integrating patient-generated data into the consultations. Furthermore, we analyzed interviews with all participants to study how patient-generated data enhance patient-provider communication (66 interviews in total; physicians were interviewed once per phase). In the interviews, the participants reflected on their experiences throughout the field study. The authors and the project team developed the interview guides based on the kernel theory (ie, patient-centered care). The interview guide further included questions about the participants’ experience using PatientHub and their implementation of the therapy plan. There were separate interview guides for patients and physicians and initial and follow-up consultations. English translations of the interview guides can be found in Multimedia Appendices 1-4. The interviews were held either in Swiss German or German by the lead author and experienced project members with digital health backgrounds. They were transcribed verbatim and anonymized. The combination of video recordings and interviews provided us with a rich data set to evaluate the impact of PatientHub on patient-provider communication behaviors. Furthermore, evaluating in the field offered us valuable insights into the experience of patients and physicians when engaging with PatientHub.

**Figure 8:**
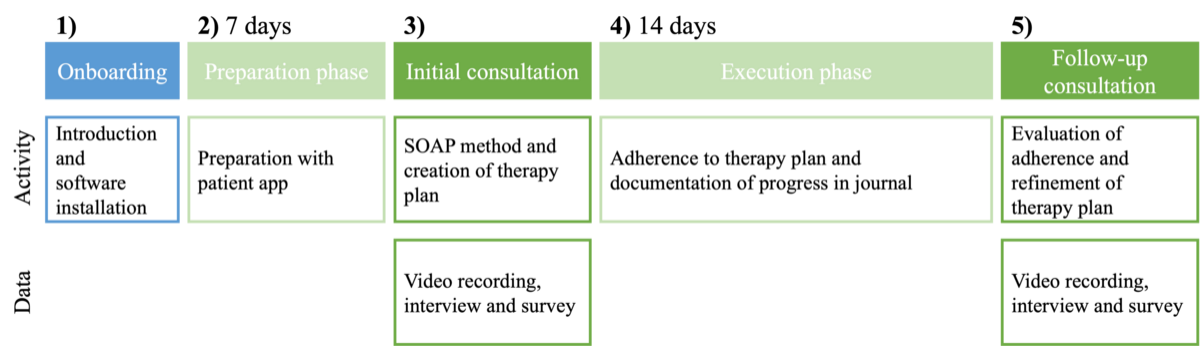
Field study design. SOAP: Subjective, Objective, Assessment, Plan.

The 3-week field study comprised five phases: (1) onboarding, (2) preparation phase, (3) initial consultation, (4) execution phase, and (5) follow-up consultation. Patients were asked to engage with the patient app of PatientHub during the preparation and execution phases. In the following sections, we outline the study design in detail, depicted in Figure 8.

#### Onboarding

##### Patients

The study team explained the study design to the patients, including the process for the 3 weeks and the study aim, and answered any questions. All patients were interviewed and completed a survey regarding their previous experience in chronic care. The patients installed and logged into the system with help from the study team. Finally, they received instructions for the upcoming week, the so-called preparation phase.

##### Physicians

The physicians were introduced to the study design and goal. They received training on PatientHub before the initial consultations. While the training focused on the consultation app, we also introduced the patient app to the physicians.

#### Preparation Phase (Patients Only)

Patients kept a journal regarding diet and physical activity as daily notes in the patient app. They were asked to complete a general health questionnaire and select 3 favorite dietary and activity interventions.

#### Initial Consultation (Patients and Physicians)

The goal of the initial consultation was for the patients and physicians to create a therapy plan with dietary and activity interventions. They reviewed the patient-generated data (ie, questionnaire and journal entries). Furthermore, they discussed a desired weight loss goal and therapy duration. Finally, they created a therapy plan for the following 2 weeks. During the consultation, patients and physicians could use the consultation tool (see the aforementioned description). Physicians saw 4 to 5 patients throughout the field study, and the patient-provider matching stayed the same.

#### Execution Phase (Patients Only)

Over 2 weeks, patients implemented the therapy plan and generated data on their progress in the journal. Before the follow-up consultation, they filled out a shortened health questionnaire.

#### Follow-Up Consultation (Patients and Physicians)

The goal of the follow-up consultation was to tailor the therapy plan to the individual patients. Patients and physicians reviewed the patient’s adherence to the therapy plan together. Insights from the discussion led to adjusting the therapy plan for the next execution phase. This marked the end of the field study.

### Participants

The field study included 28 patients and 6 health care providers (ie, physicians in this study). Only 4% (1/28) of the patients dropped out after the initial consultation (patient 24), whereas all physicians completed the 3-week study. Before the field study, we conducted a pretest in December 2022 with 2 participants to uncover and resolve software bugs and flaws in the study design. The actual field study took place in 2 clinics in Switzerland in 2 rounds between January 2023 and April 2023 for logistic reasons. The first round was conducted in January 2023 with 18 patients and 4 physicians, and the second round started in March 2023 involving 10 patients and 2 physicians. We maintained the same study design and evaluated the same prototype.

To evaluate the effects of PatientHub on patient-provider communication in the most realistic setting, the inclusion criteria for patients were (1) age of ≥18 years, (2) a BMI of >25 kg/m^2^ or specific medical indications for weight loss (eg, diabetes), (3) ability to communicate in written and verbal German, and (4) ownership of a computer and smartphone and adequate handling of both. Physicians were selected through personal contacts and had to meet the following inclusion criteria: (1) licensed physicians in Switzerland, (2) experience counseling patients with overweight, (3) ability to speak German, and (4) familiar with computers and tablets in consultations. Tables 1 and 2 present the demographic data of the participating patients and physicians, respectively.

**Table 1:** Demographic data of participating patients.

Participant	Sex	Age (y)	BMI (kg/m^2^)	Occupation
Patient 01	Male	63	36.8	Electrician
Patient 02	Female	72	35.4	Retired
Patient 03	Female	81	34.4	Retired
Patient 04	Male	41	27.6	Lecturer
Patient 05	Female	72	29.3	Librarian
Patient 06	Male	84	30.8	Retired
Patient 07	Female	78	30.5	Retired
Patient 08	Male	62	31.9	Auditor
Patient 09	Male	81	32.5	Production manager
Patient 10	Female	37	46.3	Bank clerk
Patient 11	Male	65	25.7	Electrician
Patient 12	Female	55	41.5	Office clerk
Patient 13	Male	76	30.6	Musician
Patient 14	Female	84	35.6	Homemaker
Patient 15	Female	65	30.7	Retired
Patient 16	Male	58	36.8	Driver
Patient 17	Male	60	22.3	Teacher
Patient 18	Female	61	35.3	Chairwoman
Patient 19	Male	56	30.5	Lecturer
Patient 20	Male	57	36.6	Scientific assistant
Patient 21	Female	71	40.4	Retired
Patient 22	Female	58	37.6	Depositary
Patient 23	Male	66	31.1	Retired
Patient 25	Female	59	41.1	Office clerk
Patient 26	Male	53	27.2	Remedial teacher
Patient 27	Male	75	39.5	Office clerk
Patient 28	Female	65	26.7	Architect

**Table 2:** Demographic data of physicians.

Participant	Sex	Age (y)	Workplace	Discipline
Provider 01	Female	28	Hospital	Surgery
Provider 02	Female	27	Hospital	Psychosomatics
Provider 03	Male	59	Hospital	General medicine
Provider 04	Female	58	Private clinic	General internal medicine
Provider 05	Male	52	Private clinic	General medicine
Provider 06	Female	44	Private clinic	General internal medicine

### Data Analysis

**Figure 9:**
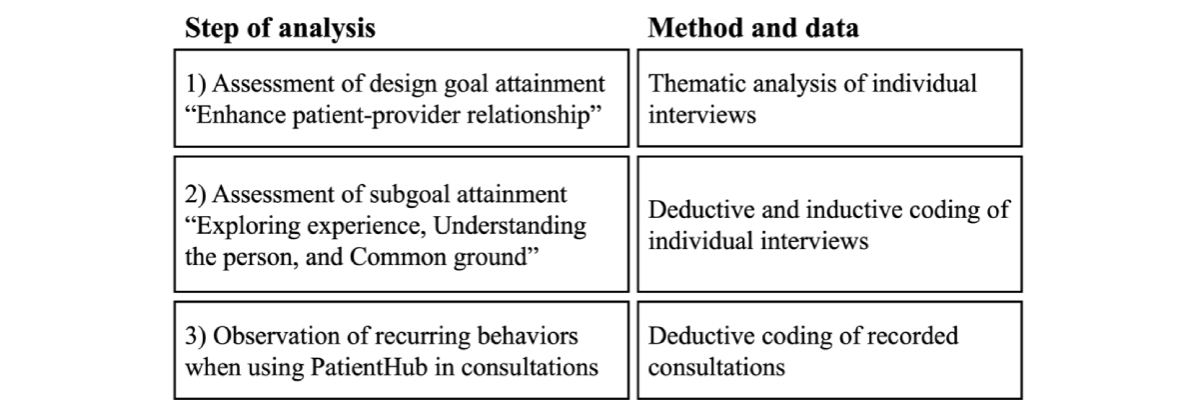
Data analysis approach.

The framework for evaluation in DSR proposes a continuum from formative to summative evaluation [55]. The framework offers 4 evaluation strategies, from which this study adopted the “human risk and effectiveness” strategy as the addressed problem is social and user centered [55]. Our analysis consisted of summative and formative elements as the study aimed to improve on the process under evaluation (ie, patient-provider communication). Accordingly, we applied deductive and inductive coding methods in 3 steps for the data analysis, as depicted in Figure 9.

To study the effect of PatientHub on patient-provider communication, we analyzed individual interviews with patients and physicians and 54 video recordings of face-to-face initial and follow-up consultations (27 recordings each). This three-step analysis allowed us to assess (1) whether and (2) how PatientHub enhances patient-provider communication (interviews) and to (3) observe recurring behaviors when engaging with patient-generated data in consultations (video recordings). The research team consisted of a graduate student with a medical and information systems background (coding author), a PhD student in digital health (lead author), and 2 senior researchers in DSR.

First, we assessed the design goal attainment “enhance patient-provider relationship” by analyzing the interviewees’ accounts regarding their perception of their relationship with the physicians (summative). After determining whether PatientHub had enhanced the patient-provider relationship, the coding author conducted a thematic analysis of the interviews [56]. This allowed us to identify the aspects that characterize high-quality patient-provider communication when using PatientHub (formative). The coding author created in vivo codes focusing on the effects on patient-provider communication attributed to patient-generated data in the consultation. The lead author conducted quality assurance by reviewing and revising the codes. In an iterative process, the lead and coding authors then grouped the codes into 4 characteristics of high-quality patient-provider communication: *personalization*, *actionability*, *trustworthiness*, and *equality*.

Second, we assessed the attainment of our subgoals “exploring experience,” “understanding person,” and “common ground” by applying a mixed deductive and inductive analysis [57,58]. We developed an initial coding scheme from related work on our kernel theory, patient-centered care [4,20]. We complemented the coding scheme with codes derived from the literature on patient-provider communication [22,23,38,39,59]. Again, the coding author created in vivo codes to capture emerging phenomena. She coded approximately 20% of the interviews before discussing the results with the lead author to refine the coding strategy. The coding author then finished coding all interviews. Finally, the lead author reviewed and refined the coding by discussing discrepancies with the coding author. In a workshop, the author group synthesized how PatientHub improved each dimension of patient-centered care.

Third, we deductively analyzed the video recordings regarding emerging behaviors [58]. The coding author analyzed the recordings based on the coding scheme applied and refined in the interview analysis. The coding scheme sensitized us to patient-centered behaviors enabled through PatientHub. During the coding of the videos, the coding scheme was expanded with in vivo codes to include emerging phenomena [58]. The video recordings were analyzed in 2 rounds on-site at one of the clinics (to ensure data privacy). We extracted emerging behaviors from the first coding round by drawing sequential processes. Initial drafts of these processes were discussed in a workshop including the author group and members from the project consortium. In the second coding round, we iterated on all video recordings based on the identified behaviors to refine our understanding and transcribed relevant sequences from the recordings. This step allowed us to formalize the 3 communication behaviors described in the *Results* section.

### Ethical Considerations

While the ethics committee of the canton of Zurich confirmed that this study is not subject to the Swiss Human Research Act (BASEC-Nr. Req-2018-00847), we still decided to obtain written consent from all patients and physicians before the field study. The informed consent form educated participants about their rights and responsibilities, data use, and privacy measures following the World Medical Association Declaration of Helsinki [60]. The participants had the possibility to opt-out at any time during the study. The form also informed the participants’ data will be gathered deidentified by assigning each participant with an identifier (ie, patient 1 to patient 28 and provider 1 to provider 6). Multimedia Appendix 5 provides the authors’ positionality statement to give the readers a better understanding of the authors’ backgrounds.

The participating clinics selected patients using a purposeful sampling approach [61]. The compensation for patients was CHF 50 (approximately US $60) and a raffle ticket for a restaurant voucher worth CHF 200 (US $230.15).

## Results

### Overview

In this section, we present our findings. First, we examine the attainment of the design goal (ie, enhance the patient-provider relationship) and discuss the 4 characteristics of high-quality patient-provider communication identified in the analysis. We then examine how patients and providers perceived patient-centered care in these consultations (ie, subgoals). Finally, we introduce 3 emerging communication behaviors when engaging with patient-generated data.

### Enhancing the Patient-Provider Relationship With PatientHub

#### Overview

When asked about their relationship with the providers, the patients reported high satisfaction with their interaction compared than previous experiences. They often described an intimate connection that would feel significantly older than the 3 weeks of the valuation. In total, 4 central characteristics emerged from our data analysis that explain this enhanced patient-provider communication. Patients and providers commonly raised these characteristics when asked about their perception of the new approach supported by PatientHub compared with previous experiences. In the following sections, we explore each characteristic in more detail. Textbox 1 provides quotes from patients and providers for each characteristic.

**Characteristics of enhanced patient-provider communication with exemplary quotes.**

**Personalization of health care**

- “She has tried to address the fact that I am not allowed to be overburdened with walking because the knees just do not work” (patient 07). Others highlighted the enriching discussions they had with the physicians “because I noticed that these are not standard answers.... He looks at you [the patient] as a human being, as an individual” (patient 25).

**Actionability of interventions**

- “It is simply a clean data basis. Now we’re talking facts and not ‘How did you perceive it?’ or ‘How was it for you?’ but Bam! There! Suddenly, ‘how many times did you go on the cross trainer?’ ‘how many times did you get the interval fasting done?’” (patient 04).
- “I was really happy to see the results. Because I remember the last time you said that you do so much, and you don’t see any results, and now we have the result” (provider 01).

**Trustworthiness of communication**

- “She was prepared. So, she read my brainy entries [laughs].... So, she obviously prepared for me. She looked at the questionnaire that I filled out [during the preparation phase]. She wrote down questions about it. That really feels good” (patient 17).
- “They were unbelievably more trusting. They revealed so many, many things. So, I think it was a very different level of trust already compared to last time” (provider 05).

**Equality of partners**

- “I came here prepared and I already had ideas. If I had to choose favorites now [in the consultation], I would have come and I would have accepted [the physician’s proposal]. Then you are externally steered” (patient 26).

#### Personalization of Health Care

Patient-generated data support patients and providers in personalizing health care as they facilitate in-depth discussions about the patients, their context, and their experience with their health. The data provide a solid foundation based on facts instead of gut feelings and memory. Therefore, the mutually agreed upon therapy plans consider the patient to be a person with their preferences, needs, and limitations. The physicians were understanding when proposing interventions and considered the patients’ circumstances. All patients in the interviews highly appreciated this (Textbox 1).

#### Actionability of Interventions

Patients and providers appraised the concreteness of the discussions facilitated by the consultation tool with the intervention screens (Figure 4, right). Many patients reported previous frustrating experiences in which providers stayed abstract in their recommendations (eg, “eat less sweets”). Due to the more integrated and holistic understanding of the patients, providers and patients could concretely discuss problems and priorities, goals, and expectations regarding each other’s roles. Many specifically highlighted how the patient-generated data allowed them to agree on actionable interventions. Therefore, patients perceived providers as more empathic and engaged in their health journey. For example, patient 26 “felt joy from the provider.... I think she was very motivating and also praised that I had done well.” The providers proved the perception right as many were pleased with their patients’ progress, such as provider 01 and patient 02 (Textbox 1).

#### Trustworthiness of Communication

Patients and providers believed that sharing patient-generated data requires trust in the first place and creates trustworthy communication. Patients perceived it as appreciation (Textbox 1, patient 17). The providers reciprocated this appreciation. When asked about the relationship between her and the patients after only 2 consultations, provider 05 highlighted how PatientHub created a trusting foundation that made the discussions in the consultations much more meaningful (Textbox 1, provider 05).

Approximately half (15/27, 56%) of the patients raised the topic of surveillance concerning sharing their data. However, most patients appreciated the subtle surveillance as it made the therapy plan more binding. Only 19% (5/27) of the patients felt uncomfortable sharing too much personal information. Accordingly, they only shared what they felt comfortable with in the journal.

#### Equality of Partners

Finally, PatientHub leads to a shift in the perceived roles of patients and providers. Many patients perceived control over the decisions made in the consultation. This perceived control leads to the feeling of cooperation between equal parties in the decision-making process. The patients felt strengthened in their position as they were the experts on their data (Textbox 1, patient 26).

The preparation allowed patient 26 to have an opinion instead of mindlessly accepting the provider’s proposition. Furthermore, in the follow-up consultations, patients defended their standpoints and argued for changes to the therapy plan. For example, patient 18 demanded the reintroduction of carbohydrates into her diet due to her physically demanding job. As a result of this approach, patient 23 experienced the consultation as “an open conversation and not somehow top-down. On the same level and friendly.” Many providers, too, remarked on the shift in power balance.

### The Process of Patient-Centered Care

Our analysis elicited the 4 characteristics of high-quality patient-provider communication. In the following sections, we explore the process of enhancing patient-provider communication by discussing the 3 dimensions of patient-centered care (ie, subgoals)

#### Finding Common Ground

Overall, we observed that the consultations centered on the 3 aspects of finding common ground: problems and priorities, goals, and roles. PatientHub introduced patient-generated data into the natural consultation process through screens for goal setting, interventions, and planning. Patients and providers reported that the tool was a significant part of the consultation as it formed the starting point for exploring problems, priorities, and roles. The data also served as a reference to argue for or against a proposition, thereby shaping the individual roles. Therefore, patients and providers arrived at mutual decisions regarding all 3 aspects of finding common ground. When asked about the reasons for the positive impact of PatientHub, provider 01 answered the following:

You are pulling in the same direction. And are in the same reality. And that makes a much better team. And just have a more balanced, I do not want to say power balance, but a more balanced decision-making.

However, patient-generated data did not solely support finding common ground directly. Each behavior explored the other 2 components to indirectly inform mutual decisions made in the consultations.

#### Exploring Health, Disease, and the Illness Experience

The general health questionnaire and journal entries allowed patients and providers to discuss the patients’ unique perceptions of their health in both consultations (ie, the “exploring experience” subgoal). For example, provider 02 recognized in the journal overview (Figure 7, right) that patient 08 ate too few vegetables and drank too much alcohol, which the patient agreed with. Instead of staying abstract about the consumed amount of alcohol, they had a clear impression of the number of alcoholic beverages that the patient drank in a week. Several patients realized during the consultation that they were emotional eaters. The patient-generated data prompted the provider or patient to highlight such experiences. In addition to behaviors, they often discussed emotional aspects of the patient’s experience. For example, 2 patients said that they did not like swimming because they did not want to show themselves in bathing suits. Patient 11 mentioned in the questionnaire that he feared the health problems associated with obesity. During the consultation, provider 03 could follow-up on this answer by asking why the patient was afraid. Patients and providers explored the target weight reported in the questionnaire during goal setting. Often, they discussed the origin of this specific target, such as a feeling of well-being or a historic weight they had during a significant part of their life (eg, before they became parents).

Patient-generated data had an even more profound impact during the follow-up consultation. Patients and providers could gather an integrated understanding of the patients’ experience in the execution phase. The patients documented their emotions and thoughts using emojis, pictures, and text in journal entries (Figure 6). These data provided patients and providers with a rich foundation for discussions in the follow-up consultation. Instead of relying on the patients’ memory and accuracy, providers had in-depth insights into the adherence and patient experience. Sometimes, patients highlighted a journal entry because they believed that it was significant for their (lack of) success in following the therapy plan. For example, patient 07 referred to the picture of an icy peer to explain her nonadherent behavior to the “walking” intervention. Most providers emphasized the benefit of recording a patient’s emotional state. This way, they could inquire about negative feelings related to a specific intervention. For example, provider 01 could identify a potential correlation between patient 01’s emotional state and his adherence to intermittent fasting. Together, they explored that patient 01 was under pressure at work during the execution phase. This led to him feeling tense and not sleeping well. Therefore, he was not motivated to adhere to intermittent fasting. However, they realized that the patient indeed felt better on days when he could adhere to the intervention, as provider 01 recalled in the interview:

And then you could break that down nicely and say, hey, you did it. The mood was good. Look at the app. It was ALWAYS good for you to do [intermittent fasting]. And then it really came back from the patients like this: Yes, that’s right.

#### Understanding the Whole Person

Traditionally, consultation time is limited to a few minutes per patient. This limited time often does not allow providers to ask questions not directly associated with the presented problem. Consequently, understanding the whole person often falls victim to other, more pressing matters. However, the journal entries and the corresponding overview and filters allowed providers to understand the patient’s daily life (“understanding person” subgoal). In addition, the journal entries served as the foundation to further gain a better understanding of the whole person during the consultation (Figure 7). For example, provider 03 and patient 12 discussed her consumption of vegetables, where patient 12 said the following:

Probably too little in proportion. Because I have to be honest, I don’t like to cook..... And many times, it is so my partner works irregularly. And when we come home in the evening, something should just quickly be on the table. And I don’t want to stand two hours in the kitchen when I have worked all day.

This quote illustrates how patient-generated data prompted provider 03 to learn about patient 12’s experience and her proximal context—her partner working shifts might interfere with regular habits. In general, the available information and the subsequent discussion yielded interesting insights that providers usually would not obtain, as they all said during the interviews. For example, it became evident that chocolate yogurt was a central piece of patient 18’s diet. Patient 28 preferred to walk alone as he was an only child. Patients 01 and 25 had dogs, but another household member usually walked them. Patient 03 cooked for her husband and did not think he would want to eat less meat or try different grains. In addition, she drove him to therapy and, therefore, had less time for cooking.

In general, patients believed that their data helped providers obtain a better understanding of them. For example, patient 16 liked that the provider had more background information before the initial consultation. Patient 10 shared this opinion as she believed that it would be impossible to obtain such a deep understanding in such a short time. The providers agreed with the patients and explicitly mentioned PatientHub’s advantages to understand the whole person better. Providers 01 and 04 referred to “look beneath the patient’s surface” as a significant advantage.

### Emerging Patient-Provider Communication Behaviors

#### Overview

**Figure 10:**
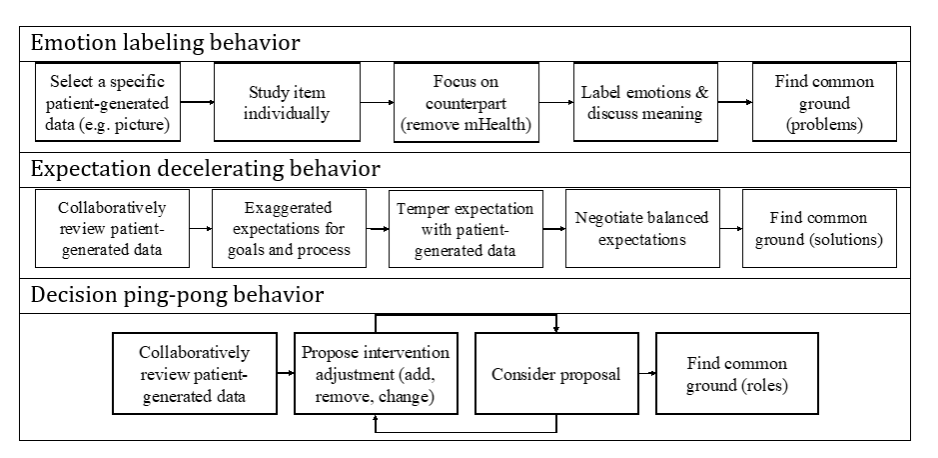
Visual representations of the 3 emerging patient-provider communication behaviors.

In the following sections, we outline 3 behaviors that we could repeatedly observe across consultations when using PatientHub. First, we describe each behavior. Then, we provide examples of the behaviors and highlight how patients and providers perceived them. Figure 10 provides visual representations of the sequential activities of the behaviors.

#### Emotion Labeling

This behavior is called emotion labeling as patients and providers discussed patients’ experiences based on patient-generated data and attached a label to it (Figure 10). The behavior occurred at the beginning of each consultation (initial and follow-up). Patients and providers sat around the table’s edge so that both had visual access to the tablet facing them. The tablet showed the journal overview with patient-generated data (Figure 7, right). The 2 variations of this behavior differed depending on the person who initiated the behavior. Providers commonly selected specific patient-generated data to start the behavior (eg, a journal entry or a questionnaire response). Often, providers referred to pictures shared by the patients. They were picked due to the client’s reported adherence or nonadherence, emotional state, or uploaded pictures. Less often, patients initiated the behavior by referring to a specific item that represented a problem or priority. The initiator pointed to the specific item and sometimes opened the journal entry. After both acknowledged the item, they moved the focus from the tablet to the other person, adjusting their seating position and turning their bodies and heads toward one another. The provider even moved the tablet out of the shared interaction space in some consultations. Then, patients and providers labeled the patients’ emotions regarding the matter represented by the selected item. They discussed the item and its meaning for the patient’s experience or context to arrive at a common understanding of the patient’s problems and priorities.

One example of emotion labeling is patient 18, who craved chocolate yogurt. The photos and text in the journal allowed provider 04 to elicit the issue and sensibly raise the topic of the large amount of sugar in chocolate yogurt. During the discussion, they explored this craving to discover 2 reasons for it. First, chocolate yogurt was a fast and enjoyable meal after a long and stressful workday. Second, the yogurt satisfied an emotional need as it was something to look forward to. Therefore, provider 04 proposed reducing the quantity of chocolate yogurt and adding plain yogurt with fruits or jam as a low-sugar alternative. This resonated well with patient 18:

Ah, I’m allowed to eat chocolate yogurt (laughs). I noticed right away that she was not telling me: You’re not allowed to do that anymore; from now on, there’s only this and that. It’s often restrictions that make things difficult. I have seen that I should lose weight over a long time, and then it doesn’t have to be as radical. This way, we can mix it up.

Patients also raised issues on their own. Patient 02 believed that one of her core problems was drinking too much alcohol, which she documented in the journal. During the interview, provider 01 explained that the journal helped her assess the quantity as unproblematic and that she would have reacted differently without the patient-generated data. They identified the guilt associated with drinking alcohol as a constant stressor.

#### Expectation Decelerating

Expectation decelerating (Figure 10) occurs when one party needs to reduce the momentum that the other is exhibiting. The behavior followed the emotion labeling behavior, where patients and providers mutually agreed on the patients’ problems and priorities regarding weight management.

Expectation decelerating varied between the initial and follow-up consultations. In the initial consultation, the providers changed the topic to discuss target weight. They reviewed the patient’s desired weight and therapy duration before the consultation. However, they first invited patients to talk about their target weight and the reason for this specific target. Upon understanding the patient’s reasoning, they focused on the goal-setting screen (Figure 4, left), where the patient-generated data are prefilled (ie, target weight and therapy duration). Providers proposed an intermediate goal and explained their reasoning depending on the targeted weight loss and therapy duration. To illustrate the proposal, providers moved the weight and time slider to the proposed goal. A scale icon indicated the sustainability of the target weight and time frame on a color scale from green to red. A 500-gram weekly weight loss was considered sustainable [62]. If the chosen target was beyond this limit (ie, the scale icon was in the red area), the providers would highlight this and explore the relationship between weight and time with the patient. They simulated different scenarios to understand sustainable weight loss. In the end, they left the decision up to the patients. The exploration led to either a prolonged therapy duration or setting an intermediate goal.

In the follow-up consultation, expectation decelerating occurred after reviewing the patient’s adherence using the journal entries. To conclude the emotion labeling behavior, patients or providers often proposed adding new interventions or increasing their frequency (eg, running more often). While substituting ineffective interventions usually generated mutual agreement, adding to the existing interventions was often more intensely debated. Frequently, the providers advised against a patient’s request, referring to patient-generated data to highlight the continued effectiveness of the existing interventions. However, patients also had to decline similar propositions by providers. Similarly, they referred to their journal entries or insights based on these data to argue their position. The following excerpt shows a discussion regarding the therapy duration in the initial consultation between patient 06 and provider 02:

Provider 02: I have seen your target weight or desired weight would be 76 kg.

Patient 06: I am also already satisfied with 77 to 78 kg.

Provider 02: So, let’s say we want to aim for 78 kg (sets target weight with slider). What is your wish when you want to reach it?

Patient 06: Yes, that’s what I am like. I want it as soon as possible. But then you gain it back again soon, right?

Provider 02: Yes exactly. So, what does soon mean concretely? We can now play it through virtually what that means as a weekly goal. So that would be 4 kg if we say 78 kg. When do you want to have that?

Patient 06: I don’t know, I say in two months it should be possible.

Provider 02: In two months, that would be eight weeks (Provider adjusts the slider on the tablet). That is not completely unrealistic for 4 kg, but it is relatively strict. So, if you actually don’t want it to be an extra burden, an additional task, then it’s too ambitious. Then I would rather put it at three months, right? (adjusts slider). Then you see, there is the scale that shows how much weight you would have to lose or how ambitious that it is. We are now well in the green zone. Concretely that would mean a little more than 300 grams decrease per week.

Often, patients entered the consultation with overambitious goals, as they recalled themselves in the interviews. Furthermore, they were enthusiastic after the 2-week execution phase as they could follow most interventions and proposed including more interventions. Providers used patient-generated data to decelerate the patients’ drive to maintain sustainable progress and prevent setbacks. While deceleration might be negatively connotated, most patients drew motivation from this behavior. For example, patient 02 mentioned that the 5 kg they decided on motivated her more than if she had tried to lose the initial 15 kg. Furthermore, patient 12 summarized the following:

Yes, and what really stuck with me was that he took a little bit of the pressure off. When you always have the feeling that you have to lose as much weight as possible as quickly as possible. That he then said: It’s good and healthy to lose 500 grams a week. That has stayed with me very much, and has also motivated me, so that I did not think: Oh in 14 days I should be 10 kg lighter.

Patient 16 agreed and mentioned the sliders, visualizing the relationship between weight loss and time, as essential for setting realistic goals. The providers agreed with this perception as the patient-generated data would provide a solid foundation to assess the therapy plan’s effectiveness, which is usually missing in conventional consultations. Provider 06 highlighted the following:

Yes, I always have the feeling that I always have to make suggestions. And [with the patient-generated data] you could say, we’ve done a good job. We are good. We continue to do so. Done.

Provider 02 agreed that patients usually quit because they want to achieve overly ambitious goals. With patient-generated data, they could credibly show that it is possible to lose weight with small and sustainable changes.

#### Decision Ping-Pong

Decision ping-pong refers to the back-and-forth process of mutual decision-making (Figure 10). It occurred when creating and adapting the therapy plan. The input for this behavior was patient-generated data created outside the consultations. During the preparation phase, patients selected favorites from a list of dietary and activity interventions and documented their daily lives in journal entries.

In the initial consultation, providers initiated the behavior by moving to the interventions screen to display the patient’s favorites, marked with a “heart” icon (Figure 4, right). Providers asked the patients why they selected the specific interventions to start the decision ping-pong. Often, patients chose the interventions as they had previously engaged in an activity or started the intervention themselves only recently. Next, providers asked patients which favorites to add as an intervention. After the patients decided on an intervention, they discussed details such as frequency and duration. A back-and-forth followed to first agree on a frequency and duration (if applicable). Once agreed, patients and providers negotiated the timing of this intervention (ie, days and time of the day). While providers often proposed the frequency and duration, the patients usually initiated the discussion on timing according to their professional and private situations. This ping-pong was repeated until both were satisfied with the therapy plan. Interestingly, patients started proposing interventions themselves after some repetitions (eg, after adding 2 interventions).

In the follow-up consultation, the behavior occurred slightly differently. First, patients or providers initiated the behavior to discuss the necessity of exchanging ineffective interventions based on the journal entries crafted by the patients in the execution phase. The behavior ended if they mutually agreed not to adjust the therapy plan. If they decided to adjust, the behavior continued as in the initial consultation.

The following excerpt shows a discussion between patient 10 and provider 03 about an activity intervention:

Patient 10: Or aerobics would be something I would like to do.

Provider 03: So once in the evening?

Patient 10: Yes.

Provider 03: Thursday is busy [with other interventions]

Patient 10: Then, we will take Monday.

Provider 03: Or after cleaning (both laugh).

Patient 10: No, thank you.

Provider 03: Monday?

Patient 10: Yes.

Provider 03: In the evening?

Patient 10: Mhm.

Provider 03: How long?

Patient 10: Half an hour.

Provider 03: Half an hour. I think so too. We will just put that in now.

A total of 30% (8/27) of the patients explicitly mentioned their appreciation for the realistic goals as an outcome of this process. Patient 21 liked that the provider said that “You cannot just cancel everything overnight. Then you just stop again.” After 2 weeks, patient 16 compared the results to other diet regimes he had followed in the past:

The whole thing is calmer and less stressful. It feels easier. It goes on for a longer time, but it is more pleasant to get through the day that way.

The provided selection of tasks and the talk with the physician helped produce ideas that the patients usually would not have produced. Patient 04 said that he would not have chosen intermittent fasting but he did because a professional explained it. The decision ping-pong showed that it could be adapted to his situation. The process also inspired the providers as provider 05 said that he would not have all these ideas spontaneously.

## Discussion

### Principal Findings

Supporting patient-provider communication using patient-generated data is a growing topic of interest in health informatics and related fields. However, the existing literature often overlooks the processes (ie, communication behaviors) when enhancing patient-provider communication using patient-generated data. More importantly, existing mHealth apps are seldom integrated into the provider’s workflow and do not sufficiently engage and activate patients in patient-centered care [44-46]. Through a design science approach, we explored how to design an integrated digital health system and its impact on the communication behaviors of 27 patients and 6 providers.

Previous work has demonstrated the benefits of integrating patient-generated data into consultations [32,35,38,45,63,64]. We expand on this work by studying the effect of patient-generated data on patient-provider communication behaviors. As argued in the Introduction section, current research focuses on the input (patient-generated data) and output (enhanced patient-provider communication; Figure 2). Our analysis elicited 3 emerging behaviors that open the black box of patient-provider communication. On the basis of our theoretical foundation and empirical findings, we propose patient-centered communication in 3 design principles to operationalize the patient-centered care framework.

### Facilitate Emotion Labeling to Explore Health, Disease, and the Illness Experience and Understand the Whole Person

The emotion labeling behavior highlights how the input is initially processed. Patient-generated data enable patients and providers to reflect on patients’ emotions related to their health. For example, patients speak about their insecurities when engaging in physical activities in public due to being overweight. Prompted by photos, emojis, and text, patients and providers explore patients’ unique experience with their health and disease (“exploring experience” subgoal). Furthermore, emotion labeling uncovers obstacles in the patients’ journeys to better health. As described in the Results section, journal entries allowed patient 03 and provider 01 to uncover and discuss the reason for the patient’s nonadherence. Patient-generated data allowed the patient and provider to understand the patient as a whole person and their proximal context (“understanding person” subgoal).

Existing research has shown how visualizations of health data increase health literacy in patients [38]. Our results show how patient-generated data are used to not only educate patients but also collaboratively generate new insights. We argue that patient-generated data enhance patient-provider communication by stimulating the exploration of a patient’s experience and context. Providers can usually only scratch the surface of a patient’s story in consultations. PatientHub introduces them to their patients’ world, allowing them to dive below the surface and gain an integrated understanding. The patients themselves also profit from their data. Our results show that generating data often initiates self-reflection that fosters patients’ understanding of themselves. Therefore, patient-provider communication becomes more patient centric. Patients and providers explore the patient’s experience and context to find common ground regarding problems and goals.

### Facilitate Expectation Decelerating to Find Common Ground on Problems and Solutions

The expectation decelerating behavior processes patient-generated data differently. Instead of beginning with a patient’s experience or context, patients and providers used patient-generated data to assess problems or goals. Our results show 2 areas in which patient-generated data decelerated patients and providers: setting goals and developing therapy plans. Providers referred to these data in the former to decelerate the patients’ ambitions. They suggested either more long-term planning or a more achievable intermediate goal. When developing therapy plans, patients and providers resisted the urge to add more or exchange interventions too quickly due to patient-generated data (“common ground” subgoal). As seen in the Results section, patient 01 could successfully argue his standpoint on keeping intermittent fasting in his therapy plan due to his data. The data allowed them to assess the therapy plan’s effectiveness, resulting in the mutual decision not to adapt it if the effectiveness persisted. Existing research demonstrates the value of patient-generated data for problem-solving and decision-making [23,64]. While we agree with their findings, the saying “It’s a marathon, not a sprint” highlights the importance of expectation decelerating. Our results show how patient-generated data are applied in consultations to resist the urge to hurry long-term behavior change. Accordingly, expectation decelerating might counteract the common pressure of a quantified self to always strive for more [65]. Quantified humans use data to continuously find inefficiencies and improve on those, often resulting in unhealthy pressure. Instead of expanding the therapy plan, patients and providers could assess the effectiveness of the current plan in the follow-up consultation. They had a reliable foundation for decision-making instead of hampering long-term success with impulsive actions. They defined achievable targets with a personalized therapy plan tailored to the patient. Therefore, patients might have more endurance in their marathon race to change their lifestyle.

### Facilitate Decision Ping-Pong Between Patients and Providers to Find Common Ground on Their Roles

Decision ping-pong reflects on how patient-generated data affect the dynamics of patient-provider communication. Existing research in medicine and human-computer interaction postulates the importance of shared decision-making [4,17,21,23,53]. Our results expand on these insights by examining how patient-generated data include the patient in decision-making as an expert and how this changes the consultation dynamics (“common ground” subgoal). Many patients noticed how their position had changed in the initial consultation compared with their previous experience. We argue that introducing patient-generated data goes beyond facilitating informed decision-making. With these data, patients provide “proof” of their adherence, for example, through pictures. Moreover, they have a much deeper knowledge of PatientHub’s content. It contains patient-generated data and evokes memories that might not be documented in the system. Equipped with this knowledge, patients become experts in their own domain: their health journey.

In shared decision-making, the implicit understanding is that providers assess the patient’s state and offer options [17]. Existing research often sees patient-generated data as crucial information for providers to offer better health services [22,64]. Studies explore ways to design technology to make large amounts of patient-generated data consumable for providers [23]. Implicitly, this focus places the responsibility for data interpretation on the providers. However, we believe that patient-generated data could partially relieve providers from this burden. Patient-centered care emphasizes mutual decisions; therefore, patients should carry this burden with their providers.

Our results show that most patients willingly accepted such a role as they felt taken more seriously, could defend their position, and perceived having control over the decision-making process. Integrating patient-generated data into the consultation empowers patients to assume a more active role in their health care. We argue that providers must not be solely responsible as our results highlight how patients become experts in their health journey. Instead, the responsibility is shared between patients and providers according to their expertise. While providers assess the data against their medical knowledge, patients interpret the data in the context of their lives and experiences. Therefore, their communication becomes truly “respectful of and responsive to individual patient preferences, needs, and values and ensuring that patient values guide all clinical decisions” [16].

### Conclusions

This study evaluated communication behaviors that emerge when introducing patient-generated data into patient-provider communication. We studied how these behaviors in patient-provider communication actualize the potential of patient-generated data to increase patient-centeredness. Our analysis uncovered 3 communication behaviors in medical consultations when using PatientHub. Furthermore, we demonstrated how enhanced patient-provider communication is necessary for patient-centered care. On the basis of our findings, we believe that this study contributes to research in 2 ways. First, we emphasize the value of patient-provider communication. The identified behaviors demonstrate how data-supported patient-provider communication creates value, not the technology and data themselves. Second, the behaviors offer actionable insights into implementing patient-centered care.

However, this study does not come without limitations. First, while we evaluated PatientHub in the most realistic setting, its applicability in the real world depends on regulatory and security frameworks. Second, the generalizability could be further increased with a larger sample size and a randomized controlled trial. Future research could also investigate how patient-generated data empower patients individually to study the changing role dynamics in medical consultations.

## Appendix

Multimedia Appendix 1 Interview guide: physician initial consultation (English translation from German).

Multimedia Appendix 2 Interview guide: patient initial consultation (English translation from German).

Multimedia Appendix 3 Interview guide: physician follow-up consultation (English translation from German).

Multimedia Appendix 4 Interview guide: patient follow-up consultation (English translation from German).

Multimedia Appendix 5 Authors’ positionality statement.

## Abbreviations

DSR: design science research
mHealth: mobile health
