# Supplementary material for: How Patient-Generated Data Enhance Patient-Provider Communication in Chronic Care: Field Study in Design Science Research
Source: JMIR Med Inform. 2024 Sep 10;12:e57406. doi: 10.2196/57406 (PMC11422739; doi:10.2196/57406)
Supplement: Multimedia Appendix 1 [file medinform_v12i1e57406_app1.docx]

## Interview Guide – HCP (after initial consultations)

*Please note: Original interview guide is in German. The guide is divided into subtopics with main questions and follow-up questions (in case participants would not touch upon the topics). The aim of the interviews was to let the participants narrate as much as possible.*

| **Features in Scope** | | | |
| --- | --- | --- | --- |
| Journal (daily note)   - Photos - Notes - Emotions - Filter option | Preparation Questionnaire   - Answer - Measures favorites | Knowledge Encyclopedia   - Contents - Structuring   - Definition   - Details   - Graphics/Videos | Planning   - Goal - Movement - Nutrition - Planning (calendar) |

**Consultation preparation:**

- How did the information provided by the patient help you prepare for the consultation? (🡪 Features )
- What do you think about accessing this information or presenting it through the consultation tool? (NOTE: Questionnaire is printed for HCP, but can access it) (🡪 Features )
- Would they, and if so, how, use the consultation tool to prepare for consultations? (🡪 Features )
  - How do you usually prepare for consultations? What is your working process?
  - What worked/didn't work with the tool? What is missing? (🡪 Features )
  - How would this affect your working hours overall? Prolonging, shortening, constant?

**Initial Consultation:**

- How did you feel about the initial consultation?
- How did the *consultation preparation* affect your consultation? What is different than usual? What is better, what is less? (🡪 Features )
  - What aspects of the preparation have played a role in this?
- How did the *consultation tool* affect your consultation?  What is different than usual? What is better, what is less? (🡪 Features )
  - What aspects of the tool played a role in this?

**Therapy implementation**

- What would be your prognosis regarding the patient's adherence to therapy?
  - Consideration of patient preparation
  - Consideration of consultation tool and patient app

**Knowledge Dictionary (if time)**

- What do you think of the concept of the knowledge lexicon? (*Note: If the HCP do not remember, only briefly refer to the training, otherwise omit questions*)
- What benefits do you see in this?
- What do you think of the structuring in details/definition/graphics/videos?
