# Supplementary material for: How Patient-Generated Data Enhance Patient-Provider Communication in Chronic Care: Field Study in Design Science Research
Source: JMIR Med Inform. 2024 Sep 10;12:e57406. doi: 10.2196/57406 (PMC11422739; doi:10.2196/57406)
Supplement: Multimedia Appendix 2 [file medinform_v12i1e57406_app2.docx]

## Interview Guide – Patients After Initial Consultation

*Please note: Original interview guide is in German. The guide is divided into subtopics with main questions and follow-up questions (in case participants would not touch upon the topics). The aim of the interviews was to let the participants narrate as much as possible.*

**Consultation preparation:**

| **Features in Scope** | | |
| --- | --- | --- |
| Journal (daily notes)   - Photos - Notes - Emotions | Preparation   - Questionnaire - Measures favorites | Knowledge Encyclopedia   - Contents - Structuring   - Definition   - Details   - Graphics/Videos |

- Can you tell me how this week of preparation for the first consultation was for you?
  - What was particularly good? What was rather bad/difficult/tedious?
  - How did you feel about it?
  - How did the patient app help you prepare for the initial consultation? (🡪 Features )
    - Which aspects of the app have helped you in particular?
    - What else would you have wished for in pre-season?
    - What do you think about the questionnaire you filled out on the patient app in preparation for the consultation?
    - How did you find the opportunity to document your everyday life and state of mind with regard to the initial consultation?
    - How did you find the opportunity to shoot or upload photos?
    - How was the selection of favourites for exercise and nutrition measures for you?
    - To what extent did the further information behind the "i" help them?

**Initial Consultation:**

| **Features in Scope** | | | |
| --- | --- | --- | --- |
| Journal (daily note)   - Photos - Notes - Emotions - Filter option | Preparation Questionnaire   - Answer - Measures favorites | Planning   - Goal - Movement - Nutrition - Planning (calendar) | Consultation setting (seating arrangement, shared screen) |

- How did you feel about the initial consultation?
  - What did you particularly like about the consultation? (🡪 Features )
  - Were you able to follow the doctor well during the consultation? Better than in previous consultations? Why? (🡪 Features )
  - How does this consultation differ from other consultations you have had in the past?
  - What was better and worse about this consultation compared to your previous experience? (🡪 Features )
  - What impact did the preparation have on the consultation?
- How did the information from the consultation preparation flow into the consultation?
  - How did you feel when discussing the information?
    *Prompts: Shame, pride, interest, etc?*
  - How did the doctor respond to this information? (🡪 Features )
  - How were you able to use the information in the consultation?
  - How else could this information have been used?
- How did the development of therapeutic measures go for you? (🡪 Features )
  - How did you feel about it?
  - What role did the consultation tool play in the agreement of nutrition and exercise measures? Why?
    - What did you particularly like about the tool?
    - What other possibilities would you have expected? Why?
  - What did you think was good?
  - What do you think is missing?
  - In your opinion, who made the decision on the measures? Why?
    *(NOTE: shared decision-making, ownership, etc.)*
    - Why were you (not) able to make the decision on your own?
    - How satisfied are you with the decision-making process?
  - How satisfied are you with the therapy plan that you have developed for the doctor? Why?
  - How did the information from the preparation influence the preparation of the therapy plan? *(NOTE: Here we want to deal specifically with the influence of preparation (i.e., self-observation during a week)*

**Therapy implementation**

- What are you most looking forward to in the next two weeks? Why?
- What are you LESS looking forward to in the next two weeks? Why?
- Why did you choose the confidence value? NOTE: Check the survey
- What additional support will you be looking for?
  *Prompt: from people or tools/apps such as calendar reminders?*
