# Supplementary material for: How Patient-Generated Data Enhance Patient-Provider Communication in Chronic Care: Field Study in Design Science Research
Source: JMIR Med Inform. 2024 Sep 10;12:e57406. doi: 10.2196/57406 (PMC11422739; doi:10.2196/57406)
Supplement: Multimedia Appendix 3 [file medinform_v12i1e57406_app3.docx]

# Interview Guide HCP Follow-up Consultation

*Please note: Original interview guide is in German. The guide is divided into subtopics with main questions and follow-up questions (in case participants would not touch upon the topics). The aim of the interviews was to let the participants narrate as much as possible.*

| **Features in Scope** | | | |
| --- | --- | --- | --- |
| Journal (Daily Note & **Agreement Notes**)   - Photos - Notes - Emotions - Performance - Filter option | Preparation Questionnaire   - Answer - Comparison between questionnaires 1 and 2 | Knowledge Encyclopedia   - Contents - Structuring   - Definition   - Details   - Graphics/Videos | Planning   - Goal - Movement - Nutrition - Planning (calendar) |

**Consultation preparation:**

- How did you use the consultation tool to prepare for the consultation? Did you use it differently than in the first consultation? (🡪 Features )
- How helpful do you find the consultation tool for consultation preparation? What do you like, what do you dislike? Was something missing? (🡪 Features )

*NOTE: Prepare by looking at what the HCPs said in preparation for the initial consultation*

**Followup Consultation:**

- How did you use the consultation tool in the follow-up consultation? (🡪 Features )
  - What features did you use?
  - What worked well? What less?
  - Was something missing?
- What changes (or can change) in the consultation through such a tool? Why? *Prompt: ask for negative and positive changes* (🡪features )
- How did the consultation preparation affect your consultation? What is different than usual? What is better, what is less?
  - How did you move from looking back (journal entries) to adapting therapy? Was that natural? How could this be done differently?
- How did the consultation tool affect the follow-up consultation?  What is different than usual? What is better, what is less? (🡪 Features )
- Does your role change during the consultation, and if so, how?
  - Were you (HCP) different?
- How did you experience the patients during the follow-up consultation? Differently? How different? Why?
  - What differences have you observed between the initial and follow-up consultation?

**Therapy implementation**

- Has your prognosis regarding therapy adherence been confirmed from the first consultation?
- What are your impressions of your patients' adherence to therapy (compared to consultations outside of evaluation)?
  - How do you assess the influence of the consultation tool and the patient app on therapy adherence? Which elements have which influence?
- What are your impressions of your patients' health literacy (compared to consultations outside of evaluation)?
  - How do you assess the influence of the consultation tool and the patient app on health literacy? Which elements have which influence?

**General and final**

- What do you personally take away from participating in this evaluation? Why exactly that?
- Where do you see potential for the consultation tool and the patient app for medical therapies used in the consultation?
  - How could this change the *cooperation* between patient and doctor?
  - How could this change the *(trust) relationship* between patient and doctor?
  - How could this change the *empowerment* of patients?
  - How could this change the *health literacy* of patients?
  - How could this  *change patients'* self-confidence?
  - How could patients*' self-efficacy change*? NOTE: HCP may not know the term
  - How could patient *adherence* change?

**Definition Self-Efficacy:**

*Self-efficacy describes the conviction of being able to carry out actions on the basis of one's own competences. It is developed very early and is needed again and again throughout our lives to maintain or restore our health*
