# Supplementary material for: How Patient-Generated Data Enhance Patient-Provider Communication in Chronic Care: Field Study in Design Science Research
Source: JMIR Med Inform. 2024 Sep 10;12:e57406. doi: 10.2196/57406 (PMC11422739; doi:10.2196/57406)
Supplement: Multimedia Appendix 4 [file medinform_v12i1e57406_app4.docx]

# Interview Guide Patient Follow-up Consultation

*Please note: Original interview guide is in German. The guide is divided into subtopics with main questions and follow-up questions (in case participants would not touch upon the topics). The aim of the interviews was to let the participants narrate as much as possible.*

**Intro**

- If we start by thinking back to the first day after the consultation: How did you feel there? What stuck with you the most?
- Did you use the consultation information in the patient app after the consultation? How? For what? Why?
- How confident did you feel that day with regard to the implementation of the therapy? Why/what led to this confidence?

**Experiences with the app and therapy implementation**

| **Features in Scope** | | | | | |  |
| --- | --- | --- | --- | --- | --- | --- |
| Journal  (daily note & **agreement notes**)   - Photos - Notes - Emotions - Performance | Kons Preparation   - Questionnaire | Consultations (doc) | Calendar | Knowledge Encyclopedia | Dashboard | |

- How have you been doing since the last consultation on the implementation of therapy?
- How well did you adhere to the agreed therapeutic measures? What was easy, what was difficult? Why?
  - How did the first consultation influence the implementation of therapy? (🡪 Features )
  - What influence did the possibility of documentation have on the implementation of therapy? (🡪 Features )
    - What aspect of the documentary did you like the most? Which one is the least?
  - What influence did your knowledge that the doctor can see your journal entries have on the implementation of the therapy? *NOTE: Commitment, pressure, stress?*
- How did the patient app help you implement the therapy? (🡪 Features )
  - Regarding the entry of entries: Was it fun, was it tedious or did it go by itself?
  - How did you use the patient app to implement the therapy?
    - How did you document the implementation of your therapeutic measures in the journal?
    - When did you document the implementation in each case?
    - Are you interested in further information on medical terms or therapeutic measures? If yes/no, why? If so, have you used the relevant information in the app? How? What do you think about that? If not, why?
  - From your point of view, what was different in the implementation of therapy with the patient app compared to your previous experiences?

**Collaboration with physician in followup consultation**

| **Features in Scope** | | |
| --- | --- | --- |
| Go through the consultation preparation questionnaire | Go through the journal | Planning   - Goal - Movement - Nutrition - Planning (calendar) |

- Did you use the patient app to prepare for today's consultation (e.g. noting down questions, documenting the implementation of therapy in the journal)? If so, how and for what purpose? (🡪 Features )
- How did the second consultation go for you?
  - What did you like best? and what less? *Prompt: Is the advice more personal? How?*
  - How did your journal entries on therapy implementation flow into the follow-up consultation?
    - Are you satisfied with it? *NOTE: Is it worth keeping the journal?*
    - How did you feel during the meeting?
      *NOTE: Pride, shame, joy, etc.*
  - What impact did the journal entries have on the new therapy plan?
  - How has the consultation tool and the patient app affected your relationship with the doctor? (🡪 Features)
    *NOTE: Also address negative aspects (e.g., only focused on tablet)*
  - Was your confidence justified the day after the initial consultation? Were you too optimistic, too pessimistic or realistic? Why? *Prompt: What role did the software including the app play in this?*

**General and final (Digital Companion product)**

- What do they take away from participating in the evaluation? Why exactly that?
- **INFO:** Final questions about usefulness. We want to inquire about the usefulness of the PatientHub (i.e., the total product) to support the medical consultation.
  **Select questions to which the patient could say something:**
  - How does the tool including the app change the cooperation between patient and doctor?
  - How does the tool including the app change the (trust) relationship between patient and doctor?
  - How does the tool including the app change the patient's knowledge of the patient's state of health and illness?
  - How does the tool including the app change the self-confidence of patients?
  - How does the tool including the app change the motivation/confidence of patients?
- Do you feel better informed about the clinical picture of obesity and possible therapeutic measures than before the evaluation? If so, how does it make you feel? How does this change your relationship with the doctor (or how can it change)?
- Can you better adhere to therapy agreements with this product? *NOTE: Question refers to the overall concept*
- After the second consultation, would you use the journal differently for preparation? If so, how?
