# Supplementary material for: How Patient-Generated Data Enhance Patient-Provider Communication in Chronic Care: Field Study in Design Science Research
Source: JMIR Med Inform. 2024 Sep 10;12:e57406. doi: 10.2196/57406 (PMC11422739; doi:10.2196/57406)
Supplement: Multimedia Appendix 5 [file medinform_v12i1e57406_app5.docx]

### **Appendix – Positionality Statement**

We would like to provide the reader with a positionality statement by the authors regarding their research domain, personal context, and research approach. We confirm that each author attended all three areas with care and sensitivity. We aimed to be sensitive to potential biases.

Domain: Chronic diseases and weight management are topics of interest to two authors. One of the authors identifies as suffering from being overweight. While this might introduce bias, it also allowed the authors to relate to the study subjects’ experiences. The lead authors gained in-depth knowledge of digital health care since 2017 as part of their dissertation efforts. The coding author has a formal veterinarian education and information systems background. While this background increased our sensitivity to the domain, the authors lack formal human medicine knowledge. We collaborated with medical professionals to compensate for that. The medical professionals participated in designing and evaluating the PatientHub. However, they were not involved in the data analysis to ensure their self-assessment did not interfere with the insights.

Context: The authors all live in Switzerland, where the evaluation was conducted. The lead and coding authors were born in this region. The other two authors lived in Switzerland since 2002 and 2012, respectively. This background allows the authors to uncover the nuances of cultural peculiarities from interview statements.

Research approach: The authors have over 40 years of combined experience in design science research. This research primarily focuses on design-related dyadic collaboration. Our background ensures high sensitivity for aspects relevant to this research (i.e., work practices, artifact design, and evaluation). However, it might bias our interpretation of medical consultations.
